# Constraints on muscle performance provide a novel explanation for the scaling of posture in terrestrial animals

**DOI:** 10.1098/rsbl.2013.0414

**Published:** 2013-08-23

**Authors:** James R. Usherwood

**Affiliations:** Structure and Motion Laboratory, The Royal Veterinary College, University of London, North Mymms, Hatfield, Herts AL9 7TA, UK

**Keywords:** posture, gait, scaling, locomotion, running

## Abstract

Larger terrestrial animals tend to support their weight with more upright limbs. This makes structural sense, reducing the loading on muscles and bones, which is disproportionately challenging in larger animals. However, it does not account for why smaller animals are more crouched; instead, they could enjoy relatively more slender supporting structures or higher safety factors. Here, an alternative account for the scaling of posture is proposed, with close parallels to the scaling of jump performance. If the costs of locomotion are related to the volume of active muscle, and the active muscle volume required depends on both the work and the power demanded during the push-off phase of each step (not just the net positive work), then the disproportional scaling of requirements for work and push-off power are revealing. Larger animals require relatively greater active muscle volumes for dynamically similar gaits (e.g. top walking speed)—which may present an ultimate constraint to the size of running animals. Further, just as for jumping, animals with shorter legs and briefer push-off periods are challenged to provide the power (not the work) required for push-off. This can be ameliorated by having relatively long push-off periods, potentially accounting for the crouched stance of small animals.

## 1. Introduction

Larger birds and quadrupeds tend to support their weight during locomotion with more upright, relatively stiffer limbs [1,2]. This has been attributed to the structural challenges imposed by scaling: geometrically similar forms of consistent material properties scale body weight in proportion to the cube of length, but strength scales in proportion to cross-sectional area—a square of length. More upright postures result in ground reaction forces passing closer to the joint centres, thereby reducing the externally applied moments and the mechanical loading on the supporting tissues. However, the geometric benefits of upright limbs would also apply to small animals, allowing them even lighter limbs for a given safety factor, or an improved safety factor for geometrically similar bones and muscles. To date, there has been no mechanical or energetic account for smaller animals benefitting from more crouched postures; some benefits relating to stability, manoeuvrability or control are generally suggested [1–3].

Theoretically, minimal-work walking and running require biologically unachievable, infinite, ‘impulsive’ forces [4,5]. Consider running: an exceedingly stiff, upright leg operating at a very low duty factor *β* (proportion of the stride period with the foot in contact with the ground) allows nearly vertical (albeit very high) forces, thereby avoiding the costly fore–aft fluctuations in speed. Some compromise prevents such gaits from being realized in biology. Might this compromise be that between muscle power (reduced with longer stance periods) and work (reduced with briefer stances), and would this compromise scale with size?

**Figure 1.**
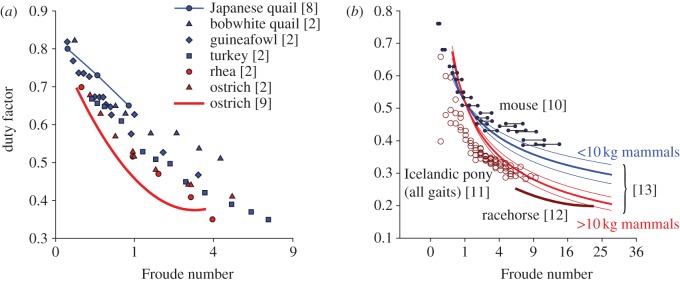
Published duty factors of avian bipeds (*a*) and quadruped forelimbs (*b*) relating to Froude number. Larger animals (above 10 kg in red) have lower duty factors than smaller animals (below 10 kg in blue) at equivalent Froude numbers. Regressions shown as bold lines. Quadruped regressions with 95% CIs (bounding thin curves) are for eight large species and three small. Mice are assumed to have leg lengths of between 2 and 3 cm (resulting in uncertainty in Froude number, denoted by the horizontal lines). See text for definition and discussion of Froude number.

**Figure 2.**
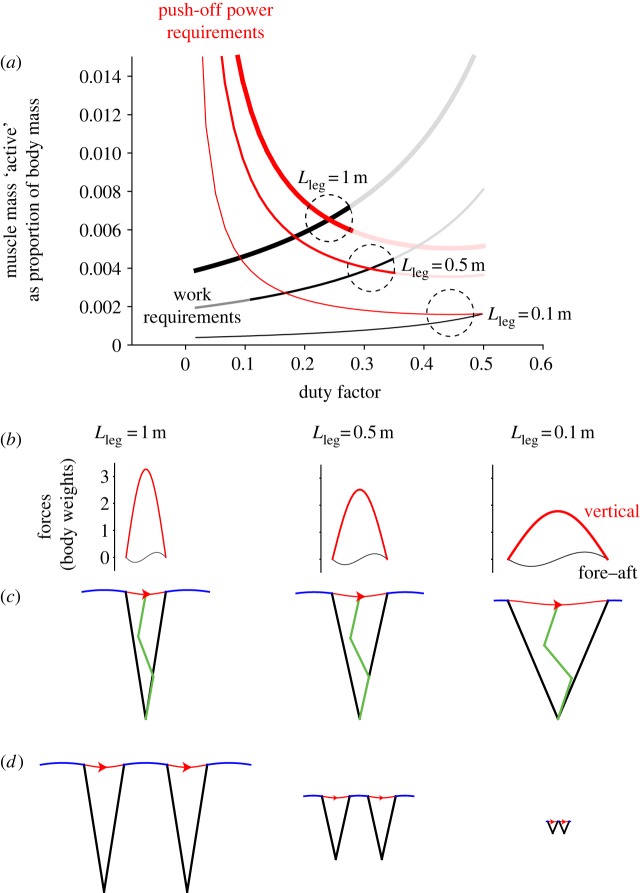
A numerical demonstration of the implications of differential scaling of work and power requirements despite approximate dynamic similarity. The active muscle demand due to push-off power (red curves) is high for very brief stances; the active muscle demand due to work (black lines) is high for long stances due to large horizontal impulses and fluctuations in kinetic energy. Larger animals (thicker curves) require greater proportional muscle activation. The consequences in terms of leg forces through stance (*b*), kinematics scaled to leg length (*c*) and unscaled kinematics (*d*) are shown for the optimal duty factors for minimizing active muscle volume (dashed circles in (*a*)). Smaller animals are predicted to use larger duty factors in order to reduce the active muscle volume required for power during push-off. Larger duty factors require greater limb compression at midstance, demonstrated by the green three-segment legs in (*c*).

Animals much below the size of a gibbon cannot put as much mass-specific energy into a leap, or jump as high (absolutely) because, to do so, their (absolutely) short legs and resulting brief take-off periods would require excessive powers [6,7]. Might a similar power constraint for small animals undergoing steady locomotion account for their high duty factors (figure 1; [2,8–12]), higher mechanical cost of transport (see the electronic supplementary material based on Heglund *et al*. [13], but contrasting with the conclusions of Biewener [1] and Heglund *et al*. [13]) and crouched postures?

## 2. Overview

This study first describes the scaling of work and power requirements for dynamically similar gaits. It then gives a numerical demonstration of the scale-dependent compromise between minimizing active muscle volume for work versus for power.

## 3. Scaling of work and power requirements for dynamically similar gaits

The principles of dynamic similarity are usually related to the Froude number, a term describing the forces associated with changing direction as a proportion of those due to gravity. The concept is familiar to marine and structural engineers and to animators: big waves, big falling skyscrapers and big dying monsters all ‘look’ big because they appear to fall slowly; that is, it takes a long time for them to fall. The principle has been valuable to biology, allowing gait mechanics to be meaningfully compared for animals across large size, speed and gravity ranges. Dynamic similarity does appear to apply—at least as a first approximation—for both bipeds and quadrupeds: small animals have to take more, quicker steps to travel at the same speed, and the walk–run gait transition occurs at similar Froude numbers for animals covering a large size range. Following Alexander & Jayes [14], objects moving with precise dynamic similarity, despite differences in size (leg length (*L*_leg_) being a convenient measure) or gravity, will:

— have identical motions or displacements—step length, height fluctuation, etc.— once scaled by leg length;
— have identical periods—whether stride, step, stance or leg swing—once scaled by the time taken for a ball to fall from a height of leg length, or a pendulum of leg length to swing. Thus, comparable periods for dimensionally similar gaits are proportional to 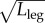; a monster four times the height of a human should take twice the time to fall over; and
— experience the same forces, once scaled by body weight (mass *m* times gravity *g*).

Therefore, normalized positive mechanical work for dimensionally similar gaits—the force (per body weight) times some deflection (per leg or step length *L*_step_)—is also identical. This is sometimes termed a dimensionless mechanical cost of transport CoT_mech_.3.1
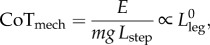
for perfectly dynamically similar gaits, where *E* is a suitable mechanical work, such as the positive ‘push-off’ work during the acceleration phase of stance. While CoT_mech_ for dynamically identical gaits is not dependent on size, the work and power requirements, even if normalized by body mass, are. The mass-specific work of each step for a precisely dynamically similar gait (so step length is proportional to leg length) of a geometrically similar (so 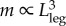) animal is, from expression (3.1),3.2
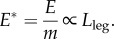
The mass-specific power during stance P* relates to the work, stride period *T* and duty factor *β*:3.3
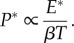
For precise dynamic and geometric similarity, the duty factor is constant, and the stride and stance period are related to pendulum or ballistic mechanics 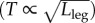. Thus,3.4
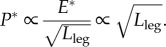
Therefore, larger animals require relatively more muscle to be active (whether for work or power) if locomoting with dynamic similarity [14]. Further, the dissimilarity in scaling for work and power requirements—just as for jumping—is revealing. Smaller animals would find providing the power proportionally more challenging than the work (expressions (3.2) and (3.4)). This may account for the more crouched postures of smaller animals, enabling higher duty factors and slight deviation from dynamic similarity to reduce the push-off power requirements (expression (3.3)), despite higher work demands owing to larger stance angles resulting in greater deceleration and acceleration.

## 4. A numerical demonstration of the scaling implications of minimizing active muscle mass required for work and power in running

The following model explores optimal (minimum ‘cost’) gaits given three assumptions concerning the fundamental properties of muscle:

— the ‘cost’ of locomotion relates, to some relevant extent, to the volume of muscle activated—not merely the mechanical work or power requirement. Physiological measurements indicate that activation accounts for a considerable portion (around 35%) of the cost of muscle contraction due to calcium pumping [15]. In the numerical analysis below, it is assumed to be the *only* cost. This is clearly wrong in detail, but is intended as a revealing alternative extreme to the assumption of *only* a cost to work, which, while successfully accounting for walking and running, predicts unrealistically high- (infinite-) force gaits [4,5]. Note that no constraints are considered in terms of muscle stress (i.e. muscle strength) or strain *per se*: it is assumed that limits due to these properties can be entirely circumvented with suitable lever arm ratios;
— a given mass of muscle can produce a limited amount of work per contraction. This is fundamentally constrained by the number of cross-bridge cycles performed; and
— a mass of muscle can produce a limited instantaneous, peak or push-off (during only the acceleration phase of stance) power during contraction. Some constraints to the biochemistry of reaction rates presumably limit the instantaneous power achievable by muscle.

For the numerical model, the muscle capability (in mass-specific terms) of both work and mean push-off power are assumed to be totally scale independent. In reality, of course, this is likely to be untrue: there are certainly differences in cross-bridge density, details of muscle biochemistry [16] and elastic recoil—this last point being of relevance to both work and power constraints [6].

The kinetics of running can be conveniently modelled (see, [17] for details) using a half sine-wave vertical force of appropriate amplitude to oppose mean body weight, and by calculating the horizontal forces required to direct the net force vector through the centre of mass. Adding the kinematic inputs for an appropriately scaled human runner of leg length 1 m, jogging at a speed *V* relating to a Froude number *Fr* of 1 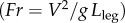 and a leg swing period of 0.315 s, allows the consequences of duty factor in terms of muscle work and mean push-off power to be calculated numerically. These are expressed as masses of muscle required to provide the work or power (figure 2). In this model, all and only the positive work and power required at the centre of mass are taken to be a result of muscle activity (no elasticity or ‘internal’ work is included). Muscle work and mean push-off power were taken as 80 J kg^−1^ muscle and 800 W kg^−1^ muscle, respectively, selected as approximate maximal values for leaping primates [7,18].

## 5. Discussion and implications

The differential scaling of work and power requirements has clear parallels with jumping: in both cases, smaller animals experience greater challenges to producing the power required during contact with the ground. In the case of jumpers, proportional elongation of legs (deviation from geometric similarity) is a clear adaptation for limiting the adverse implications of small size. In steady terrestrial locomotion, the crouched posture associated with high duty factors of smaller animals may play a similar role.

The simple running model agrees (figure 2): at *Fr* = 1, the duty factor that minimizes the mass of activated muscle increases with decreasing size; as predicted from the scaling arguments, the muscular demands for work become proportionally reduced at smaller sizes. Further, at sufficiently small sizes, work requirements stop being a consideration: the optimal duty factor is simply that which minimizes mean push-off power (figure 2, *L*_leg_ = 0.1 m).

The scaling and model therefore provide a novel account for ‘grounded running’ (high duty factor) gaits in medium and small birds, and more generally for increased crouchedness at smaller sizes; higher duty factors require the limb to undergo greater strains (table 1), and thus be relatively more flexed at midstance. Conversely, larger animals are proportionally more challenged by the work (versus the push-off power), and hence favour stiffer, more upright and more economical (see the electronic supplementary material) gaits.

**Table 1.** Model results for minimizing active muscle mass given constraints to mass-specific muscle work (80 J kg^−1^ muscle) and push-off power (800 W kg^−1^) for a runner at *Fr* = 1.

initial leg length *L*_leg_ (m)	1	0.5	0.1
speed (m s^−1^)	3.13	2.21	0.99
duty factor	0.241	0.305	0.442
leg strain at midstance (%)	3.2	4.9	11.3
work/ideal vertical work	1.75	2.10	3.37
